# Video recording and vegetation classification elucidate sheep foraging ecology in species‐rich grassland

**DOI:** 10.1002/ece3.8172

**Published:** 2021-10-20

**Authors:** Stephen J. G. Hall, David R. Arney, Robert G. H. Bunce, Elis Vollmer

**Affiliations:** ^1^ Estonian University of Life Sciences Tartu Estonia

**Keywords:** animal‐borne video, grazing behavior, plant‐animal interactions, sheep behavior

## Abstract

1. Factors influencing grazing behavior in species‐rich grasslands have been little studied. Methodologies have mostly had a primary focus on grasslands with lower floristic diversity.
2. We test the hypothesis that grazing behavior is influenced by both animal and plant factors and investigate the relative importance of these factors, using a novel combination of video technology and vegetation classification to analyze bite and step rates.
3. In a semi‐natural, partially wooded grassland in northern Estonia, images of the vegetation being grazed and records of steps and bites were obtained from four video cameras, each mounted on the sternum of a sheep, during 41 animal‐hours of observation over five days. Plant species lists for the immediate field of view were compiled. Images were partnered by direct observation of the nearest‐neighbor relationships of the sheep. TWINSPAN, a standard vegetation classification technique allocating species lists to objectively defined classes by a principal components procedure, was applied to the species lists and 25 vegetation classes (15 open pasture and 10 woodland) were identified from the images.
4. Taking bite and step rates as dependent variables, relative importance of animal factors (sheep identity), relative importance of day, and relative importance of plant factors (vegetation class) were investigated. The strongest effect on bite rates was of vegetation class. Sheep identity was less influential. When the data from woodland were excluded, sheep identity was more important than vegetation class as a source of variability in bite rate on open pasture.
5. The original hypothesis is therefore supported, and we further propose that, at least with sheep in species‐rich open pastures, animal factors will be more important in determining grazing behavior than plant factors. We predict quantifiable within‐breed and between‐breed differences, which could be exploited to optimize conservation grazing practices and contribute to the sustainability of extensive grazing systems.

## Graphical abstract

Sheep carried video cameras enabling their interactions with the vegetation of their species‐rich pasture to be evaluated. Their behavior, as measured by bite rates, depended far more on their individuality than on the vegetation. This novel combination of vegetation analysis and video technology could provide underpinning for the practice of grazing in areas of conservation importance.

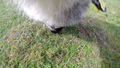

## 1 INTRODUCTION

Many landscapes of high biodiversity and cultural significance are intimately associated with traditional livestock systems (Bunce et al., 2018a, 2018b; Plieninger et al., 2015; Redecker et al., 2002). At a more local level, within areas dominated by conventional agriculture, areas of high floral and faunal biodiversity may survive, such as many of the nature reserves in the UK (https://jncc.gov.uk/) and elsewhere. Therefore, at both landscape and local levels, conservation management is required, often with support from agri‐environment schemes. Conservation grazing may be applied, which is the use of grazing livestock to promote habitat and species diversity by the removal of excess herbage and the management of vigorous, invasive species. Management is usually empirical and site‐specific (Rook, Dumont, et al., 2004) and needs to be underpinned by ecological and behavioral research on how grazing livestock exploit these landscapes.

In the global context, local breeds of livestock are often claimed to possess local adaptations, which may be highly relevant to the sustainability of pasture‐based livestock farming under conditions of global climate change (Boettcher et al., 2015). Animal genotypes that possess relevant behavioral adaptations, such as appropriate patterns of selectivity (Meuret & Provenza, 2015; Provenza et al., 2003), would therefore be important genetic resources, but the underlying genetics are incompletely understood. Specific regions of the sheep genome have been shown to have been under postdomestication selection (e.g., Fariello et al., 2014; Paim et al., 2018), but no such region directly relating to grazing behavior has yet been identified. However, it is clear from quantitative genetics that some behavioral components of grazing behavior are heritable (Fogarty et al., 2009; Snowder et al., 2001), supporting the contention that grazing behavior is an outcome of genotype–environment interaction (Osoro et al., 1999; Rook, Dumont, et al., 2004). Nongenetic animal factors such as individual learning and social interactions (Provenza & Balph, 1990; Sibbald et al., 2008) modulated by traditional herding practices (Meuret & Provenza, 2015) will also be important. As a result, there is considerable, and poorly understood, individual variation in the structure of grazing behavior (Searle et al., 2010).

There have been many studies of how sheep under extensive husbandry range among plant communities (e.g., Baumont et al., 2000; Hester et al., 1999; Michelena et al., 2009), and of the nutritional consequences (e.g., Mobæk et al., 2012), but the structure of grazing behavior in semi‐natural species‐rich pastures of conservation interest has received relatively little attention. The types of grasslands where most experimental and modeling work has been conducted have been either relatively simple swards, or mosaics, where patches of favored plants have occurred within a matrix of less attractive vegetation. Little formal experimentation on the grazing of semi‐natural pastures of conservation interest has been made (but see, e.g., Hellström et al., 2003), although some general principles exist. For example, there is evidence that plant species richness itself enhances foraging motivation, from both field studies (Arzak et al., 2016) and cafeteria feeding trials (Feng et al., 2016), suggesting the influence of vegetation on grazing behavior is not simply attributable to available biomass or to cover abundance of favored species (Parsons et al., 1994).

From a long‐term study in a species‐rich alpine grassland, Mysterud and Austrheim (2016) hypothesized that the “dietary composition” of sheep arises from a combination of “sheep preferences” and “immediate environment.” We have tested this hypothesis in a similarly diverse vegetation environment, but with a more behavioral approach. Bite and step rates were taken as surrogates for dietary composition, and the focus has been on a single component of the immediate environment, namely, the vegetation within which bites are prehended. Sheep preference is clearly a multifactorial term, which was not defined by Mysterud and Austrheim (2016). As its surrogate, we have adopted sheep identity (the serial code of each animal) a term which encompasses all morphological, behavioral, experiential, and other variables in which one sheep might differ from another. Formally, we predict that bite and step rates are influenced by the categorical factors sheep identity, vegetation, day, and time of observation. Social behavior and grazing behavior are known to interact (e.g., Sibbald et al., 2008), so we investigated whether variation in social behavior, expressed as nearest‐neighbor relationships, also varied according to vegetation.

In a novel development, we are taking as our “vegetation” term an objective multivariate assessment of the species composition of the area being grazed by the animal, without reference to the cover abundance (extent of ground covered) of individual plant species. Assessment was by a standard technique (TWINSPAN: Kent & Coker, 1992), which, though well known to plant ecologists (Hearn et al., 2011), seems not to have been widely adopted by animal ecologists. This investigation of a hypothesis therefore also evaluates the use of a novel combination of ecological and behavioral approaches.

## 2 METHODS

### 2.1 Study area and animals

The study took place on semi‐natural species‐rich neutral *Agrostis capillaris*‐*Festuca rubra* pasture with spatially variable hydrology and unfenced adjoining woodland, on a farm within Lahemaa National Park, in northern Estonia (59^o^30'N, 25^o^40'E), previously described by Hall et al. (2020). Most plant species present are not uncommon in Estonia, but the grass *Holcus mollis,* which was fairly abundant, is nationally rare (Kull et al., 2002). The woodland was not closed‐canopy and the ground flora was relatively dense, such that when observing individual sheep in this area it was often not possible to see many other flock members.

Field work took place on five days (5–9 July 2019). The weather was cool with occasional rain. The sward condition was not quantified, but there was clearly adequate forage, substantially more than in the previous year (Hall et al., 2020, and personal observation). The study flock, accompanied by four Maremmano guard dogs, in view of an apparent risk of predation from bears and wolves, was in permanent occupation and numbered approximately 50 ewes of the Estonian Native breed (Michelson, 2013). At the time of our study, the sheep were healthy, non‐pregnant, non‐lactating, and in good body condition. Sheep were handled by specialized personnel in accordance with European Union Directive No.609/1986, and the study was in full compliance with the ethical requirements of the Estonian University of Life Sciences.

The flock was housed in a barn at night and customarily foraged in an adjacent field for about 3 hr or 4 hr in the morning. Some foraging took place near the barn during the afternoon, but the main grazing period, typically of about 100 min, was in early evening when the dogs would accompany the flock across the field to a 5‐ha pasture about 250 m from the barn. Four ewes were selected on the basis of diversity of body size, as we intended to maximize between‐animal variability. Body weights were 29, 32, 42, and 46 kg and corresponding incisor breadths (measured from the marks made in a slab of dental impression material) were 29.5, 29, 32, and 34 mm. On each, a GoPro® Hero 5 Session camera (GoPro Inc., San Mateo, CA, USA) was fitted to a breast‐bone harness so as to have a field of view, while harvesting vegetation, from the forefeet to the lower forward extremity of the muzzle. The area of the field of view was therefore not standardized but remained constant for each individual sheep.

The flock was confined briefly before the morning grazing period in order to attach the monitoring equipment. After about 2 hr, the flock and dogs returned spontaneously to the barn and the equipment was removed. Sheep were confined again, the equipment was downloaded, and batteries recharged. The process was repeated in late afternoon.

### 2.2 Video, sound, and behavior monitoring

We set the cameras to record video and sound continuously. As well as providing good images of the vegetation and of steps, they also enabled registration of the bites that prehended vegetation (harvesting bites). Sounds of chewing (Galli et al., 2011) were not discerned. The data storage capacity and battery capacity of these devices enabled generation of a maximum of six time‐stamped consecutive MP4 files each of 17 min 42 s duration (total duration approximately 104 min). It was not possible to start the cameras remotely. After release, a period averaging 29 min (range 20–60 min) elapsed before the sheep had commenced feeding. Each focal (camera—fitted) sheep was watched by an observer. Our social behavior protocol was modified from that of Sibbald et al. (2005), every two minutes activity (grazing defined as prehension of vegetation, standing, walking, other behaviors) was noted. Social variables were also recorded, those used in the present analysis were the head‐to‐head distance (meters) to the nearest neighbor, estimated by eye, and whether the nearest neighbor had changed since the previous observation.

Observations were made during morning and afternoon on each of the five days. On some occasions, video records were absent or incomplete, mainly due to malfunctions of the harness or expiry of the camera battery, although behavior data continued to be collected. Observations ceased either when sheep returned apparently spontaneously to the barn, or at battery expiry. On some occasions, sheep were out of sight so only partial social behavior data were obtained.

### 2.3 Merging behavior and vegetation data

Behavior observations yielded scan data (Martin & Bateson, 1993), while the video records were continuous. Datasets were merged by selecting in each video MP4 file the image obtained at the instant of the behavioral observation. The temporally nearest bite to this time point was determined, the video paused, and the vegetation recorded from its image on a laptop screen (approximately 30 x 16 cm), rather greater than life size.

### 2.4 Vegetation

A list of species was recorded for each of the selected images, in the form of presence/absence data. In some cases, species name was assigned on the basis of apparent similarity to species that were familiar from experience in England and Scotland. In practice, none of the species thus designated were recorded in sufficient numbers to affect the results. We defined occurrence of fallen wood as a distinct species, indicating tree cover. Species richness (the number of plant species registered) was tabulated for each of the images.

When plants were only partially visible in the selected video frame, the video would be scrolled back or forwards by up to 3 s so the species could be accurately recorded. This often involved examining the area peripheral to the immediate field of view at the instant of the bite. No attempt was made to identify the actual plant species prehended. The bites and steps taken during 10 s before and 10 s after the instant of the video image were scored using Cowlog software (Pastell, 2016).

#### 2.4.1 TWINSPAN classification

Species lists were subjected to the TWINSPAN procedure in Community Analysis Package version 5 (Henderson & Seaby, 2014), which is based on an algorithm comparable with Principal Components Analysis. TWINSPAN places species lists on principal component axes and identifies the species, known as indicator species, which are the most influential in this placement. A divisive key is generated with indicator species at each division of the key. How the divisive key operates is not critical to the interpretation of our results, and we include it because reference to the indicator species helps with interpretation of the vegetation–animal relationships we have identified.

The first two axes, interpreted with reference to the known ecologies of the indicator species, enabled the underlying environmental gradient to be identified as being related to soil dampness, against a background of increasing shade. Species lists cluster on these axes, and these clusters are defined as vegetation classes. In this study, 30 of the 95 species recorded were identified as indicator species. TWINSPAN then automatically allocated each image to a vegetation class. In principle, a divisive key would identify 32 classes at the fifth division, but in this study only 25 classes were identified as some of the divisions generated classes with too few members for further classification (classes 47–49, 52–55). For some subsequent analyses, we combined classes to give composite vegetation categories. These were designated *Pasture1* (classes 32–35), *Pasture2* (classes 36–46), and *Woodland* (classes 50–63).

As vegetation classes represent relative location on a principal components axis, we defined the first axis as the x‐axis in graphical comparisons of their properties.

TWINSPAN is most familiar as a tool for characterizing the vegetation of a relatively large area. The divisive key is generated from an initial sample of relatively small plots, and this key is then applied throughout the area to be surveyed. Our procedure differed in that while the video images provided the initial sample, the resulting key was not then applied more widely. If we were to extend the study, by making a further sample from the video records, we would classify each image by using the key.

### 2.5 Statistical analysis

Statistical tests were conducted in *r* (R Core Team, 2015, Crawley, 2013). Correlations were expressed as Pearson's *r* statistic.

### 2.6 Grazing behavior

In a preliminary analysis, bite and step rates during the time period surrounding the video image were found not to be normally distributed so nonparametric statistical tests were used. Taking numbers of bites and numbers of steps during the 20 s surrounding the instant of each video image as the dependent variables, and considering images recorded in each vegetation class separately, the Kruskal–Wallis test was used to detect differences in the effects on bite and step rates of the two factors sheep identity, and day of observation. Some patterns were only discernible when composite vegetation categories (*Pasture1*, *Pasture2*, *Woodland*), rather than vegetation classes, were used, so tests were repeated on this basis.

In the 2‐min interval between observations, sheep could move from one vegetation class to another and this could influence the reliability of the assignment of bite and step rates, which were assessed over 20 s, to specific vegetation classes. Pairs of consecutive images were therefore characterized as to whether they revealed short‐term change or constancy in the vegetation classes and composite vegetation categories grazed by the sheep.

### 2.7 Social behavior

Social behavior was defined in terms of nearest‐neighbor distances, and of whether nearest neighbors of focal sheep changed during grazing. Preliminary analysis had shown that patterns were only discernible when composite vegetation categories, rather than vegetation classes, were used. We investigated social behavior in two ways, (a) at the instant of the video image, and (b) in relation to the observation 2 min previously. For (a): Nearest‐neighbor distances were compared between sheep, days, and composite vegetation categories by the Kruskal–Wallis test. For (b): Nearest‐neighbor distances (sheep and days being aggregated) were classified as either greater, or the same or smaller, than those at the previous observation. These proportions were compared in relation to the composite vegetation categories where they were observed, using the Kruskal–Wallis test. Frequencies of observations in which the sheep had changed nearest neighbor since the last observation were compared using the *χ* ^2^ test.

## 3 RESULTS

From 41 animal‐hours of video observation, 1,002 images were coincident with a behavior observation. All were used in the TWINSPAN analysis, but 27 subsequently proved unsuitable for analysis of bite and step rates, which are therefore based on the 975 images for which both rates were obtained (Table 1). Not all behavior variables could be tabulated for every image. Numbers of bites in the two main divisions (*Pasture1* with *Pasture2,* and *Woodland*) were, respectively, 22,128 and 2,357, and of steps, 7,037 and 1,645, totaled over the 20‐s periods associated with each of the 975 images.

**TABLE 1:** Durations (minutes, total 41 hr) of behavioral observations and (in brackets) numbers of video images sampled during each observation period. Of these images, 10 did not include bite and/or step data

			Sheep 1	Sheep 2	Sheep 3	Sheep 4	Totals
Day	1	AM	18 (5)	0 (0)	18 (7)	0 (0)	35 (12)
		PM	33 (5)	74 (23)	66 (24)	98 (23)	270 (75)
Day	2	AM	0 (0)	45 (20)	64 (29)	67 (34)	176 (85)
		PM	77 (24)	89 (34)	81 (33)	71 (23)	317 (114)
Day	3	AM	84 (35)	89 (37)	77 (30)	81 (32)	331 (134)
		PM	33 (8)	73 (34)	78 (39)	0 (0)	185 (81)
Day	4	AM	80 (37)	89 (40)	76 (40)	76 (36)	321 (153)
		PM	83 (40)	89 (38)	77 (33)	80 (37)	329 (148)
Day	5	AM	83 (36)	89 (32)	77 (34)	84 (29)	332 (131)
		PM	0 (0)	89 (25)	78 (29)	0 (0)	167 (54)
Totals		min	492 (190)	723 (283)	692 (298)	557 (214)	2,464 min (985 images)

### 3.1 Vegetation

Ninety‐six taxa of flowering plants, ferns, lichens, and mosses were distinguished. The mean number of species recorded per image was 4.96 (median 5, range 1–9). In addition, there was fallen wood (twigs and branches not assigned to species).

#### 3.1.1 Species richness

Species richness did not vary significantly among vegetation classes (Kruskal–Wallis test: χ^2^ = 16.81, *df* = 24, *p* = NS).

#### 3.1.2 Objective classification

**FIGURE 1:**
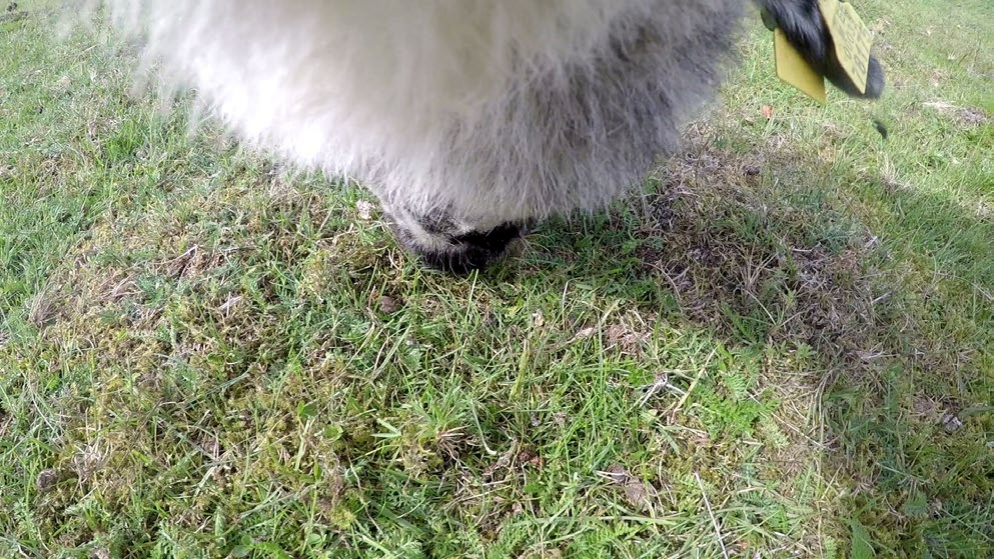
Example of video image, obtained by sheep GP1 in a pasture area. Species were scored by replaying the video for up to 3 s before and after the time signal

**FIGURE 2:**
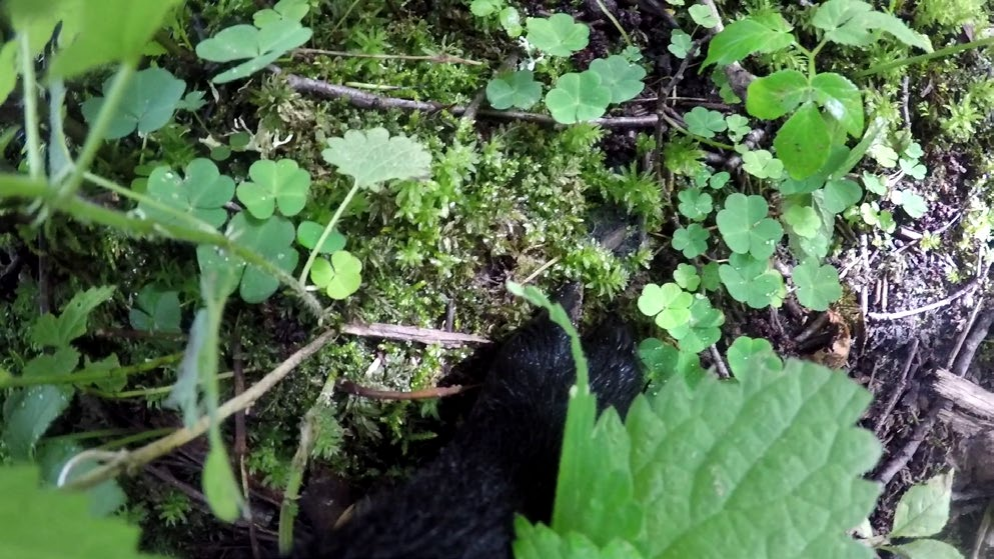
Example of video image, obtained by sheep GP4 in a woodland area

**FIGURE 3:**
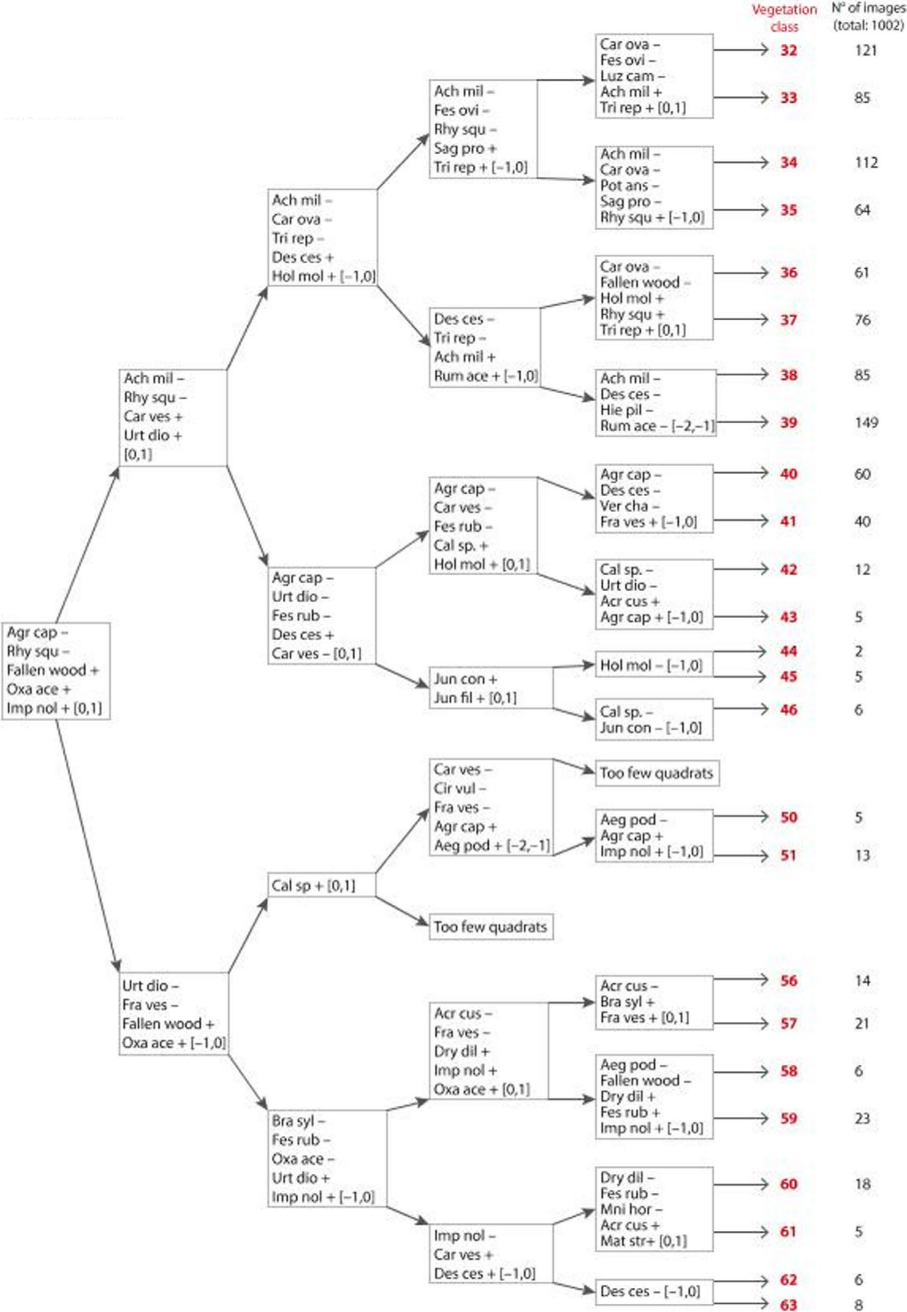
TWINSPAN classification. At each division, the relevant indicator species are listed (abbreviations: Table 1). The values in the square brackets are, respectively, the maximum indicator score for a sample to be directed upward, and the minimum indicator score for it to be directed downward. For example, regardless of what nonindicator species are present, a sample featuring the indicator species Agr cap, Rhy squ, and Ach mil would be directed upward at the first (net score −2), second (−2), third (−1), and fourth (−2) divisions, and downward at the fifth, to be placed in vegetation class 33. A sample with Fallen wood, Oxa ace and Fes rub would be directed downward at the first (net score +2) and second (+2) divisions, upward at the third (−2), downward at the fourth (+1), and fifth (+2) to be placed in vegetation class 59. Serial numbers of vegetation classes, and the number of video images assigned to each, are presented

Fallen wood and 29 species were identified by TWINSPAN as indicators. The classification, derived from 1,002 images (Figure 1 and Figure 2), is presented in Figure 3 as a divisive key, with species name abbreviations in Table 2. Vegetation classes were defined by presence or absence of indicator species. The first division in the classification directed downwards a set of 119 images of woodland character which at the second division comprised ten vegetation classes (vegetation classes 50,51 and 56–63). These formed the composite vegetation category *Woodland*.

**TABLE 2:** Abbreviations for names of indicator species

Ach mil	*Achillea millefolium*
Acr cus	*Acrocladium cuspidatum*
Aeg pod	*Aegopodium podagraria*
Agr cap	*Agrostis capillaris*
Bra syl	*Brachypodium sylvaticum*
Cal sp.	*Calamagrostis* sp.
Car ova	*Carex ovalis*
Car ves	*Carex vesicaria*
Cir vul	*Cirsium vulgare*
Des ces	*Deschampsia cespitosa*
Dry dil	*Dryopteris dilatata*
Fallen wood	Fallen branches or twigs
Fes ovi	*Festuca ovina*
Fes rub	*Festuca rubra*
Fra ves	*Fragaria vesca*
Hie pil	*Hieracium pilosella*
Hol mol	*Holcus mollis*
Imp nol	*Impatiens noli‐tangere*
Jun con	*Juncus conglomeratus*
Jun fil	*Juncus filiformis*
Luz cam	*Luzula campestris*
Mat str	*Matteucia struthiopteris*
Mni hor	*Mnium hornum*
Oxa ace	*Oxalis acetosella*
Pot ans	*Potentilla anserina*
Rhy squ	*Rhytidiadelphus squarrosus*
Rum ace	*Rumex acetosa*
Sag pro	*Sagina procumbens*
Tri rep	*Trifolium repens*
Urt dio	*Urtica dioica*
Ver cha	*Veronica chamaedrys*

Considering the 883 other images, directed upward at the first division, the second division separated 753 images of unshaded grassland (vegetation classes 32–39) of relatively nutrient‐poor character (as implied by the identification as indicator species of *Achillea millefolium* and *Rhytidiadelphus squarrosus*) from 130 images (vegetation classes 40–46) indicating a more damp and eutrophic, woodland edge environment (*Carex vesicaria*, *Urtica dioica*).

The 753 images of pasture grassland were separated at the third division into two groups. The first group (vegetation classes 32–35), numbering 382 images, was designated as composite vegetation category *Pasture1,* with relatively dry hydrology. The other 371 images (vegetation classes 36–39), which showed more shaded, damp character, were combined with the 130 images (vegetation classes 40–46) that had been separated off at the second division, to define the composite vegetation category *Pasture2*.

Characteristics of the vegetation classes are in Table 3, with the most frequently occurring species in each. Overall, the most frequently occurring species were *Agrostis capillaris* and *Festuca rubra* (723 and 711 occurrences, respectively), which occurred together in 568 of the images. For seven images, only one species was recorded. These were *Deschampsia cespitosa* (three images), *Agrostis capillaris* (two images), and *Carex ovalis* and *Oxalis acetosella* (one image each).

**TABLE 3:** Characterization of each of the vegetation classes identified by TWINSPAN (species name abbreviations are in Table 2)

Vegetation class	Composite vegetation category	Number of images	Mean (max, min) number of species per image	Total number of records of species presence	Species with more than 10% of the total number of records of presence for each vegetation class. Species are not ranked, grass species are cited first				
32	*Pasture1*	121	5.6 (9,3)	674	Agr cap	Fes rub	Ach mill	Rhy squ	Car ova
33	*Pasture1*	85	5.3 (8,2)	453	Agr cap	Fes rub	Ach mill	Rhy squ	
34	*Pasture1*	112	5.5 (9,1)	620	Agr cap	Fes rub	Tri rep	Car ova	
35	*Pasture1*	64	4.6 (7,1)	296	Agr cap	Fes rub	Tri rep	Rhy squ	
36	*Pasture2*	61	4.7 (9,2)	285	Agr cap	Fes rub	Des ces		
37	*Pasture2*	76	5.1 (9,2)	385	Agr cap	Fes rub	Des ces	Rhy squ	Hol mol
38	*Pasture2*	85	5.8 (8,4)	376	Agr cap	Fes rub	Ach mill	Rum ace	
39	*Pasture2*	149	4.6 (8,1)	686	Agr cap	Fes rub	Hol mol	Rhy squ	
40	*Pasture2*	60	4.6 (8,2)	277	Agr cap	Fes rub	Car ves	Urt dio	
41	*Pasture2*	40	4.6 (7,2)	182	Fes rub	Car ves	Urt dio		
42	*Pasture2*	12	4.6 (7,2)	63	Cal sp.	Urt dio			
43	*Pasture2*	5	5.0 (6,4)	25	Agr cap	Acr cus	Hol mol	Ver cha	
44	*Pasture2*	2	3.5 (4,3)	7	Cal sp.	Des ces	Hol mol	Ver cha	
45	*Pasture2*	5	1.6 (3,1)	7	Cal sp.	Des ces	Urt dio		
46	*Pasture2*	6	2.7 (4,2)	16	Cal sp.	Des ces	Jun con	Jun fil	
		*midpoint of classification*							
50	*Woodland*	5	5.4 (7,4)	27	Acr cus	Urt dio	Aeg pod	Fra ves	
51	*Woodland*	13	5.4 (8,4)	75	Acr cus	Urt dio	Agr cap		
56	*Woodland*	14	4.6 (8,3)	64	Fes rub	Fallen wood	Acr cus	Oxa ace	
57	*Woodland*	21	4.5 (6,3)	96	Fes rub	Fallen wood	Bra syl		
58	*Woodland*	6	4.1 (6,2)	29	Fallen wood	Bra syl	Oxa ace	Aeg pod	
59	*Woodland*	23	4.3 (7,1)	98	Fes rub	Bra syl	Oxa ace	Fallen wood	
60	*Woodland*	18	3.7 (6,2)	67	Fallen wood	Imp nol	Mni hor		
61	*Woodland*	5	3.4 (4,3)	17	Fallen wood	Imp nol	Oxa ace	Mat stru	Acr cus
62	*Woodland*	6	4.7 (6,3)	28	Fallen wood	Imp nol	Car ves	Des ces	
63	*Woodland*	8	4.5 (7,3)	36	Fes rub	Imp nol	Fallen wood	Urt dio	Car ves

### 3.2 Grazing behavior in relation to vegetation

**FIGURE 4:**
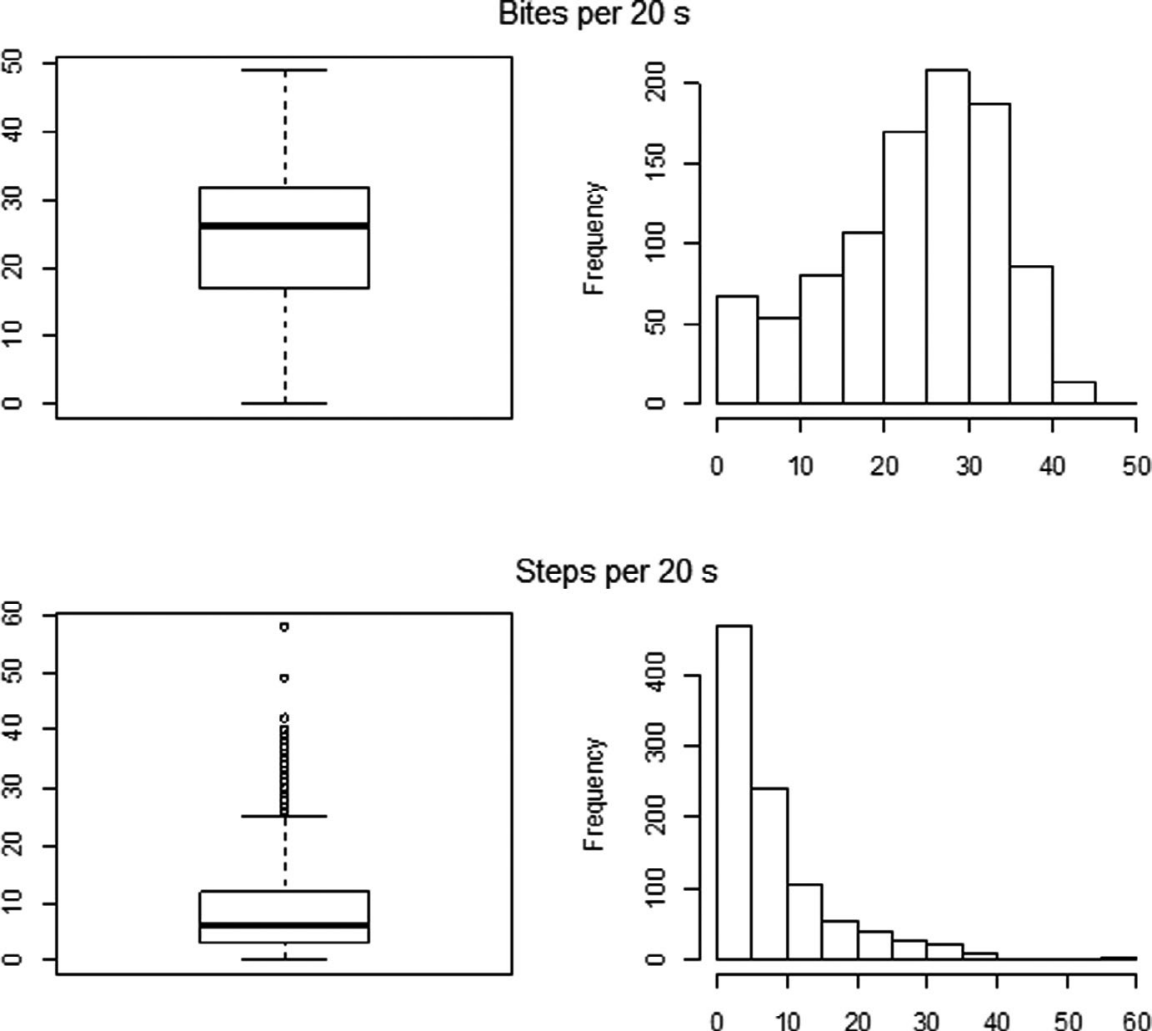
Boxplots and frequency distributions of numbers of bites and steps during the 20‐s period centered on the timing of the video image. Medians: total bites 24, total steps 6, *n* = 975. Numbers of zeros: 33, 38, respectively

Frequency distributions of bites and steps for each 20‐s period centered on the timing of the video image, over the complete dataset (*n* = 975 images with both step rate and bite rate), are in Figure 4. Overall, median numbers of bites and of steps for each 20‐s period were 26 and 6 respectively, corresponding to 78 bites/min (range 6–93) and 18 steps/min (range 6–61.5). Median bites/min for the composite vegetation categories (*Pasture1*, *Pasture2,* and *Woodland*) were 87, 75, and 31.5, respectively.

**FIGURE 5:**
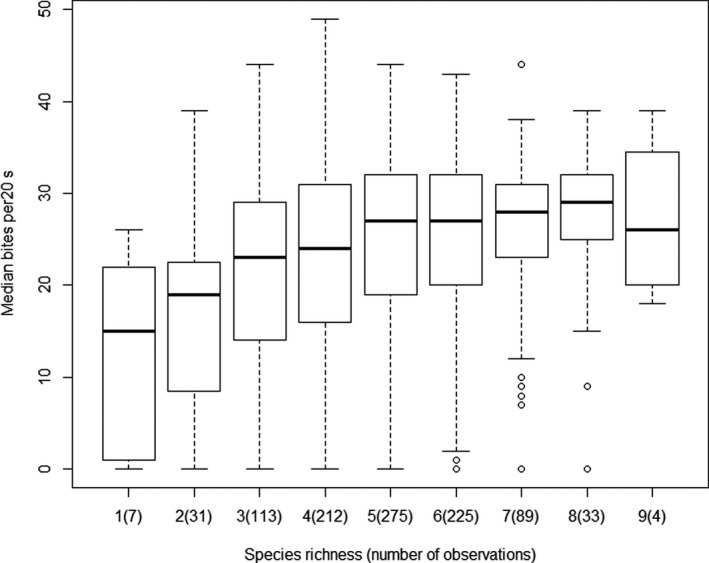
Relationship between species richness (x axis) and median number of bites per 20 s. In brackets: number of observations

Without distinguishing among vegetation classes, and considering all images for which species richnesses and step and bite rates were all available, the relationships between species richness and bite rate and step rate were deduced. The correlation was significant for bite rate (*r* = 0.204, *p* < .05, Figure 5) but not for step rate (*r* = −0.151, *p* = NS). While not statistically demonstrable, a maximal bite rate appears to be reached at the level of 5 species.

**FIGURE 6:**
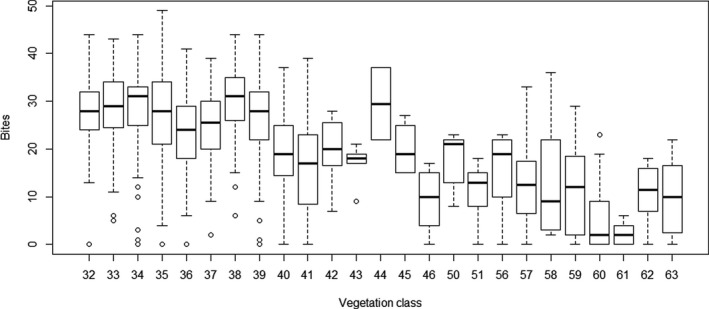
Boxplot for each vegetation class of numbers of steps recorded for each 20‐s period centered on the timing of the video image. Location of the vegetation class on the axis represents location on the first axis of the TWINSPAN ordination

**FIGURE 7:**
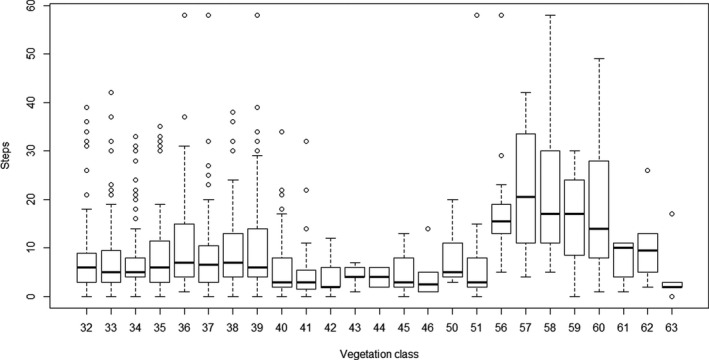
Boxplot for each vegetation class of numbers of steps recorded for each 20‐s period centered on the timing of the video image

There was an apparent relationship between relative locations of vegetation classes on the TWINSPAN ordination and bite rate, and this was less marked for step rate (Figure 6, Figure 7). Consistent with this, both bite rate and step rate differed significantly between vegetation classes (bite rates: *χ* ^2^ = 297.2, *p <* .001, step rates: *χ* ^2^ = 141.2, *df* = 24, *p <* .001, Kruskal–Wallis test). Differences among animals were less pronounced (bite rates, χ^2^ = 198.4, *p <* .001; step rates, *χ* ^2^ = 65.7, *df* = 3, *p <* .001). When only the 15 vegetation classes of a more open character (*Pasture1* and *Pasture2*) were considered, the situation was reversed. Kruskal–Wallis statistics for bite rate were 229.6 for sheep identity and 134.1 for vegetation class, and for step rate, 100.6 and 55.11, respectively, all highly significant (*p* < .001).

Bites and steps were negatively correlated for most of the vegetation classes in the *Pasture1* and *Pasture2* composite vegetation classes (Table 4).

**TABLE 4:** Variation among vegetation classes in the correlation between vegetation class and bite and step rates. Rates are expressed as the numbers of bites and steps recorded during the 20‐s period centered on each video image

Vegetation class	Composite vegetation category	Median number of bites	Median number of steps	Correlation coefficient	*n*	*p*
32	*Pasture1*	28	6	−0.209	121	<.05
33	*Pasture1*	29	5	−0.342	85	<.01
34	*Pasture1*	31	5	−0.462	112	<.001
35	*Pasture1*	28	6	−0.244	64	NS
36	*Pasture2*	24	7	−0.425	61	<.05
37	*Pasture2*	25.5	6.5	−0.119	76	NS
38	*Pasture2*	31	7	−0.224	85	<.05
39	*Pasture2*	28	6	−0.413	149	<.001
40	*Pasture2*	19	3	+0.202	60	NS
41	*Pasture2*	17	3	+0.115	40	NS
42	*Pasture2*	20	2	+0.177	12	NS
43–46	*Pasture2*	10–18	2.5–4	−0.065	12	NS
50–51	*Woodland*	21,13	5,3	−0.097	18	NS
56–57	*Woodland*	12.5, 19	15.5, 20.5	−0.672	35	<.001
58–59	*Woodland*	9, 12	17,17	−0.557	29	<.001
60–61	*Woodland*	2, 2	14, 10	−0.085	23	NS
62–63	*Woodland*	11.5, 10	9.5, 2	−0.032	14	NS

Paired comparisons of vegetation classes were possible for 875 images and the one immediately following (Table 5). In 28% of cases, during the 2 min between observations, the vegetation class being grazed remained the same. When these comparisons were on the basis of composite vegetation category, the proportion was 65%.

**TABLE 5:** Comparisons of vegetation class between pairs of consecutive video images (interval of 2 min) classified by (a) vegetation class and (b) composite vegetation category

Comparison	Number of observations	As percentage
(a) In relation to TWINSPAN classification of vegetation class		
Same vegetation class	243	28
Change in vegetation class:		
Vegetation class adjacent on classification	148	17
Vegetation class next‐but‐one on classification	101	12
More distant vegetation class	383	44
Total	875	100
(b) In relation to composite vegetation category		
Same composite vegetation category:	570	65
(Within *Pasture1*	*223*	25)
(Within *Pasture2*	*287*	33)
(Within *Woodland*	*60*	7)
Change in composite vegetation category:		
Between *Pasture1* and *Pasture2*	237	27
Between *Pasture1* or *Pasture2* and *Woodland*	68	8
Total	875	100

### 3.3 Social behavior in relation to vegetation

Social behavior data were incomplete due to animals being temporarily out of sight, and the actual numbers of observations obtained are given in the tables. Distances to the nearest neighbor (*n* = 844, range 0–20 m) varied between sheep and days, but not between composite vegetation categories (Table 6).

**TABLE 6:** Overall patterns of social affiliation: Kruskal–Wallis tests of differences among levels of the factors day, time of day, and composite vegetation category in relation to distances to nearest neighbor (NN)

Factor	Levels of factor	Number of observations	Median NN distance (m)	Kruskal–Wallis χ^2^
Sheep	1	236	1	
	2	165	2	
	3	242	2	
	4	165	1.5	60.9
	(total)	(808)		*p* <.001
Day	1	62	1	
	2	149	1	
	3	170	1	
	4	262	2	
	5	165	2	48.3
	(total)	(808)		*p* <.001
Composite vegetation category	Pasture1	335	2	
	Pasture2	406	1.5	
	Woodland	67	1	4.06
	(total)	(808)		*p* NS

When nearest‐neighbor relationships at each observation were compared with those observed 2 min previously, in 500 out of 748 observations sheep had changed their nearest neighbor, and frequencies did not differ significantly among composite vegetation classes (*Pasture1*, 205 change, 116 no change; *Pasture2*, 250, 122; *Woodland*, 45, 10, respectively; *χ* ^2^ = 2.93, *p* = NS).

## 4 DISCUSSION

We emphasize the potential value to animal ecology of well‐established objective vegetation classification methodologies such as TWINSPAN. These are used for ecological monitoring on a wide scale and are particularly suited to long‐term studies (e.g., https://countrysidesurvey.org.uk/, Hall & Bunce, 2019). In contrast, grazing studies conducted in natural and semi‐natural habitats have usually employed vegetation classification techniques which are specific to the research question being addressed. For example, in arid shrubland in Argentina, Bertiller and Ares (2008) distinguished six categories of vegetation community, which were defined in terms of cover abundance of forage species known to be preferred, coupled with morphological and phytochemical characteristics of the dominant shrub species. In northeast Scotland, Hester et al. (1999) classified heather moorland by mapping sheep paths and grass patches (the latter ranging in area between 1 m^2^ and over 200 m^2^). In some vegetation communities, direct observation of prehension of plant material is practicable (Agreil & Meuret, 2004; Cook et al., 2016; Parker et al., 1999). Sales‐Baptista et al. (2017) mounted Go‐Pro® cameras on grazing sheep, but their vegetation description technique did not focus on plant species diversity, nor did they report bite and step rates. de la Rosa (2019) used similar, but customized, equipment on a time‐lapse basis, supported by GPS, to elucidate the foraging ecology of cattle kept in extensive conditions in Mexico, recording what plant species were being eaten. There is an extensive methodology on studies in experimental swards, including multispecies grasslands, and in the laboratory where cafeteria experiments are often used to investigate forage selection. These studies have generally used very small numbers of plant species.

These approaches may not always be appropriate in the case of highly complex mosaics like our study area, which included pasture that varied spatially in relatively subtle ways, woodland, and boundary areas with at least 96 species in approximately 5 ha. Neither would they have enabled us to combine detailed behavioral studies with fine‐scale study of vegetation.

We found that differences in vegetation were highly influential on bite rates and step rates when the woodland data were included, but when only pasture areas were considered, differences among individual sheep were of greater importance than differences in vegetation, which were still, however, highly significant. Our findings are based on the relative values of the Kruskal–Wallis statistics which, in the case of bite rates exhibited on pasture vegetation, showed a greater influence of the factor sheep identity (229.6) than of the factor vegetation class (134.1). For this preliminary study, we consider a more rigorous statistical model inappropriate. In principle, variance in a behavioral phenotype such as bite rate could be partitioned between animal and vegetation factors and the relative importance of different animal factors such as within‐ and between‐breed differences could be established. The original hypothesis of Mysterud and Austrheim (2016) can therefore be refined with (using their terminology) “sheep preferences” being hypothesized as more influential on dietary composition than “immediate environment,” at least when the vegetation does not include very pronounced variation such as between woodland and pasture. This novel finding was made possible because of our combination of three methodologies, namely, video recording of the vegetation–animal interaction, objective vegetation classification with its sensitivity to relatively subtle changes in pasture vegetation, and detailed analysis of selected elements of grazing behavior.

A possible alternative hypothesis on relationships between grazing behavior and vegetation is that the sheep are responding to plant species richness per se. This can be predicted from nutritional considerations (e.g., Provenza et al., 2003). Experimental support has come from Wang et al. (2010, 2011) whose cafeteria experiments with a very diverse range of plants, supplemented by studies in sown plots, showed increased intake by sheep when more plant species were on offer, with decreasing selectivity as number of species increased, apparently to a plateau of 8 species. The correlation we found between bite rate and species richness is consistent with this alternative hypothesis. Species richness did not differ among our 25 vegetation classes, but bite rates did. As we found vegetation class and composite vegetation category to be statistically significant explanatory factors for behavioral variables, species richness and vegetation as we have classified it both appear to influence grazing behavior, possibly in different ways.

One process by which vegetation influences grazing behavior could be that, in some environments, which could in principle be identified by TWINSPAN, favored and palatable plant species are unable to form large patches because of interspecies competition or a harsher microenvironment (Grime et al., 2007). In our case, *Agrostis capillaris* and *Festuca rubra* that are both palatable to sheep could be such species. This would be consistent with the bite rates in our study (median 78 per minute) being lower than those found by other workers, on less diverse pastures. For example, Penning et al. (1991) recorded rates of 86 and 96 bites per minute from ewes (79 kg body weight) on clover and grass, respectively, while Rook et al. (2004 **)**, studying ewes (97 kg body weight) with video recording, reported 87 bites per minute on a ryegrass‐clover sward.

Bite rates are relatively easy to measure directly, but it is bite mass that is of pivotal importance in the understanding of foraging ecology (Shipley, 2007; Spalinger & Hobbs, 1992; Ungar, 1996), and the wide range of incisor breadths exhibited by our study animals will have led to corresponding differences in bite mass. While practical demonstration would be difficult, we predict that bite mass will also vary among vegetation classes.

Sheep grazing behavior is the result of the interaction of many factors, including individual variation, the behavior of other sheep, and the vegetation. Implying a prioritization of social behavior over vegetation type, Sibbald et al. (2008) interpreted this interaction in Scottish Blackface sheep on a heather‐grass mosaic, as leading sheep to favor patches of vegetation that enabled them to graze at their preferred spacing. The mean nearest‐neighbor distance in that study was 4.9 m, much greater than those observed in our study (medians 1–2 m). The close spacing evident in our study could be a breed characteristic or a consequence of management, although perceived predation risk and presence of guard dogs (Webber et al., 2015) might be influential.

Differences in bite rate among sheep were greater than differences in step rate. The relative uniformity of step rates between the vegetation classes of the *Pasture1* and *Pasture2* composite vegetation categories suggests that step rates are influenced, to some extent, by reference to the rest of the visible flock, and may indication a motivation towards flocking behavior (Dwyer, 2009). There was a negative correlation overall between step rate and bite rate consistent with the sheep maintaining step rate presumably in response to a motivation to remain close to other sheep, with their bite rates then varying accordingly. The direct relevance of bite rate and step rate to conservation outcomes does not appear to have been investigated, but Adler et al. (2001) suggested that spatial heterogeneity of vegetation would be expected to increase with patch grazing and to decrease with homogeneous grazing (in other words, to be related to the patterns of interspersion of steps and bites; Hall et al., 2020).

Classification at the level of vegetation class enabled the relative importance of animal and plant factors on bite and step rates to be assessed, but the statistically significant results in relation to social behavior mostly derive from comparisons of composite vegetation categories, which are aggregates of vegetation classes. The period of 20 s that we used could have reduced the distinctiveness, in terms of bite and step rates, of the short‐term response of sheep to vegetation differences. We consider that detailed methodological development is beyond the scope of this report, but suggest that the evident sensitivity of our approach to the mosaic structure of grasslands may well be of value.

Our finding of the substantial influence of animal individuality in relation to bite rate parallels the earlier findings of Melin et al. (2005), that 84%–98% of the variation in feeding patterns of housed dairy cows was due to individual differences. It also emphasizes how elucidating the well‐established effect on foraging behavior of animal individuality is critical to the scientific underpinning of conservation grazing (Rook, Dumont, et al., 2004). For this understanding to be applied, the genetics of grazing behavior need to be characterized (Rook, Dumont, et al., 2004). Currently, the characterization extends little further than a general understanding that sheep breeds may differ from each other in this respect, with body size being particularly important (Rook, Dumont, et al., 2004). The findings of Provenza et al. (2003) suggest that this diversity could have arisen from a diversity of grazing practices. Those based on relatively low stocking rates on pasture that is maintained at relatively high levels of available biomass may have had the effect of selecting for genotypes that graze selectively. A more episodic type of grazing at a high stocking rate would have promoted evolution for lower selectivity.

Our findings have relevance to the practical management of conservation grazing initiatives. At the technical level, the value of objective classification for assessing vegetation change has already been shown (Hall & Bunce, 2019) and practical characterization of foraging ecology could be made against this background. At the management level, this characterization could inform breeding policy within the flock or herd, or could help with the development of alternative approaches, for example specific management practices such as training animals (Thorhallsdottir et al., 1990).

There are also consequences for policymakers and for the direction of research activity. Sheep husbandry can sustain rural livelihoods but can also damage the environment and therefore poses significant challenges to policy makers (see, e.g., Bunce et al., 2018a, 2018b). While sheep grazing can promote floristic diversity (Metera et al., 2010), it can also damage sensitive species (Kose et al., 2019). A better understanding of the comparative importance of animal and of vegetation factors in modulating grazing behavior could contribute to resolving such conflicts.

On the broader scale, the sustainability, under conditions of global climate change, of pasture‐based livestock farming may depend on the availability of animal genotypes that possess relevant behavioral adaptations. Development of new breeds for sustainable commercial exploitation of rangelands is conceivable, and in such a situation, artificial selection could be applied to behavioral components of the grazing process (Beausoleil et al., 2012). Indeed, breeding for behavior traits may be preferable to breeding for anatomical adaptations. For example, selecting for bite mass, which is strongly associated with incisor arcade breadth, would also tend to select for body size (Milner et al., 2000). A change in body size could be undesirable for commercial and husbandry reasons, and selection for a behavioral trait could avoid the consequences of unfavorable genetic correlations of this kind.

Quantitative field studies of foraging ecology, in association with objective vegetation classification, have potential to elucidate the adaptability to extensive husbandry of livestock breeds. Use of appropriate genetic resources, whether as pre‐existing breeds or as the result of breeding programs within current commercial breeds, could contribute to the sustainability of extensive husbandry and to the conservation of biodiversity in species‐rich and semi‐natural pastoral agroecosystems.

## CONFLICT OF INTEREST

None declared.

## AUTHOR CONTRIBUTIONS

**Stephen J. G. Hall:** Conceptualization (lead); Formal analysis (lead); Investigation (lead); Methodology (equal); Validation (lead); Visualization (lead); Writing‐original draft (lead); Writing‐review & editing (equal). **Robert G. H. Bunce:** Conceptualization (supporting); Investigation (supporting); Methodology (supporting); Validation (supporting); Writing‐review & editing (supporting). **David R. Arney:** Conceptualization (supporting); Funding acquisition (lead); Investigation (equal); Supervision (equal); Validation (supporting); Writing‐review & editing (supporting). **Elis Vollmer:** Conceptualization (supporting); Data curation (equal); Funding acquisition (equal); Investigation (equal); Project administration (lead); Resources (equal); Validation (supporting); Writing‐review & editing (supporting).

## Data Availability

Data are in the Dryad Digital Repository https://doi.org/10.5061/dryad.t76hdr81z.
